# Primary squamous cell of the thyroid–an abbreviated clinical presentation

**DOI:** 10.1186/1916-0216-43-17

**Published:** 2014-06-18

**Authors:** Justin T Lui, Moosa N Khalil, Shamir P Chandarana

**Affiliations:** 1 Division of Otolaryngology–Head & Neck Surgery, Department of Surgery, University of Calgary, 602-1403 29 St NW, Calgary, AB T2N 2 T9, Canada; 2 Department of Pathology, University of Calgary, Alberta, Canada

## Abstract

**Background:**

Lacking any squamous epithelium, thyroid gland with primary squamous cell carcinoma (PSCC) proves to be an etiopathophysiological quandary. Two major theories do exist, though few cases have been documented to support either. We present a case that supports the “metaplasia” theory, which serves to enhance our understanding of a disease that carries with it a very poor prognosis.

**Case presentation:**

We present a case of an extremely advanced, primary squamous cell carcinoma of the thyroid with distant metastases in a thirty-six year-old male. Dying of airway compromise seventeen days following his admission, this is the shortest median survival of all documented cases.

**Conclusion:**

In addition to being the most abbreviated time period between presentation and death of all documented thyroid primary squamous cell carcinomas, we share the fifth case of thyroid PSCC in the setting of lymphocytic thyroiditis. This case should build awareness of the aggressivity of the disease and the lack of established diagnostic criteria.

## Background

Comprising less than 1% of all thyroid cancers, primary squamous cell carcinoma (PSCC) of the thyroid is exceedingly rare. It has a median age of presentation falling in the fifth and sixth decades of life [1,2]. A foreshortened history of an enlarging mass, pain, and hoarseness are often associated with presentation [3]. Diagnosis can often be challenging since squamous metaplasia is evident in other primary and metastatic thyroid neoplasms [4,5]. Moreover, the possibility of direct extension from adjacent SCC of the larynx contributes to the diagnostic difficulty [5]. The diagnosis of PSCC should include deduction via clinical, endoscopic, radiographic, and histologic inference [2,4,5]. Irrespective of multimodality treatments including surgery, radiotherapy, and chemotherapy, PSCC carries a poor prognosis. Median survival rates as short as three months have been reported [3,4].

We describe a case of an extremely advanced, primary squamous cell carcinoma of the thyroid with distant metastases in a thirty-six year-old male. With a one-month history of lymphocytic thyroiditis prior to presentation, the patient died of airway compromise seventeen days following his admission. Furthermore, given a previous diagnosis of lymphocytic thyroiditis prior to presentation, we present one of the few documented cases of PSCC in the setting of lymphocytic thyroiditis.

## Case presentation

A thirty-six year-old male presented to the emergency department with a one-month history of increasing left neck mass accompanied by pain, severe shortness of breath, and eight kilograms of unintentional weight loss. He had spent several months in Ethiopia prior to presenting to our centre, where the working diagnosis for his neck mass was lymphocytic thyroiditis, based on several FNA biopsies. As a result, propylthiouracil (PTU) and propranolol were prescribed. His past medical, family and social histories were all unremarkable for malignant risk factors. Upon presentation, he was found to be euthyroid (TSH = 0.42 mU/L, Free T3 = 4.3 pmol/L, and Free T4 = 22.7 pmol/L). His thyroid indices prior to starting PTU were unavailable and thus, we can only assume that he was in a hyperthyroid state prior to administration of PTU.

**Figure 1:**
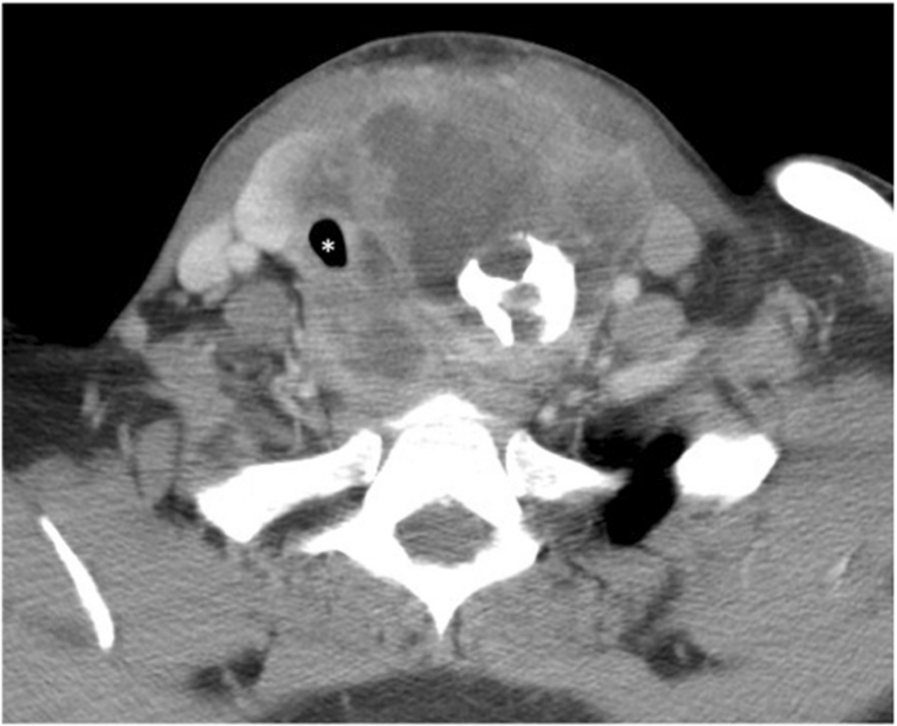
**Computer tomography (CT) of the neck revealing a 9** **×** **12 cm extensively infiltrative mass.** * = trachea.

CT scans revealed a mass arising from the thyroid measuring 8 × 9 × 12 cm, which stretched from the hyoid bone to the level of the innominate vein. The neck mass was situated just left of midline within level VI. Along with invasion of the esophagus, left thyroid and arytenoid cartilages, the tumor completely effaced both the left internal carotid and internal jugular vessels (Figure 1). The trachea was significantly displaced to the right with the narrowest luminal segment measuring 7 mm. In addition to the extensive lymph node involvement from level IA to the supraclavicular fossa, upper mediastinal lymphadenopathy with innumerable pulmonary metastases up to 2.1 cm was noted. Following the biopsy and in light of the imaging, the tumor was staged T4b, N1b, M1 [6].

In order to secure this precarious airway, an awake intubation in the operating room was successfully carried out. This was followed by an open biopsy of the neck mass. Endoscopy was not pursued given the significant esophageal obstruction. After careful review by the multidisciplinary Head and Neck Oncology team, it was felt that based on the significant invasion of neighbouring structures, the tumor was surgically unresectable. Moreover, a tracheostomy was considered unreasonable given the impressive superimposition of the tumor on the trachea. Chemotherapy and radiotherapy were considered less desirable options since the patient was now requiring aggressive ventilator and hemodynamic supports.

Given the few alternatives, a family meeting was held and as per the patient and his family’s wishes, a trial of extubation was attempted. It was agreed upon that it would not be beneficial to the patient to reintubate, should the airway deteriorate post extubation. Despite his extubation to heliox and the use of epinephrine nebulizers, he became increasingly stridulous and hypoxic. As per his previous wishes, the patient was provided comfort measures and he passed shortly after.

## Pathologic findings

**Figure 2:**
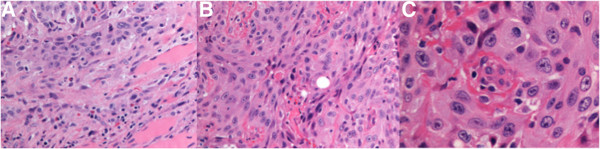
**H&E stains at 200X. (A)** Carcinoma infiltrating skeletal muscle. **(B)** Squamous cell carcinoma with polygonal cells and spindle shaped cells. **(C)** Under 400X magnification, polygonal cells with well-defined cytoplasmic borders, intercellular bridges and prominent pleomorphic nuclei were evident. A mitotic figure at 1 o’clock position is seen.

**Figure 3:**
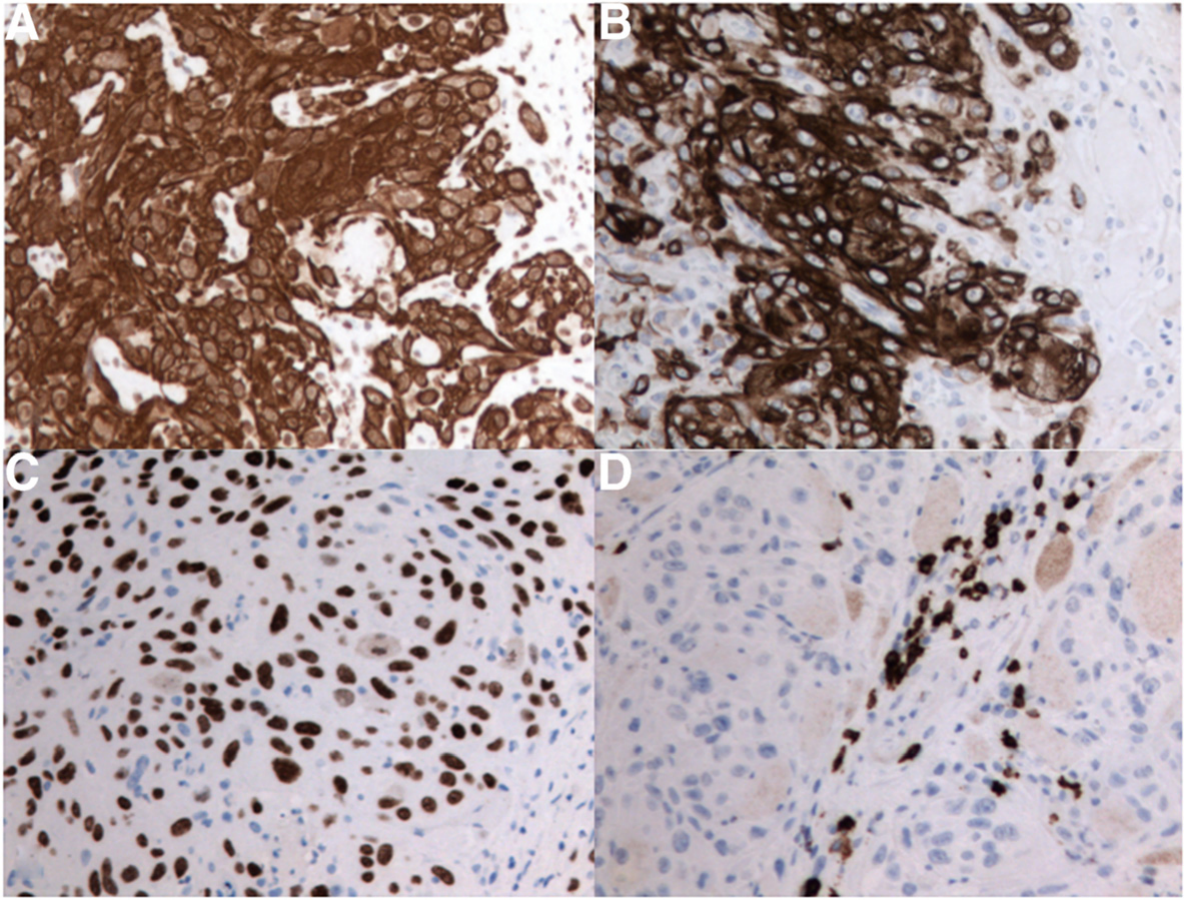
**Immunoperoxidase stains at 200X magnification. (A)** Tumor cells positive for high molecular weight cytokeratin 5/6. **(B)** Tumor cells positive for cytokeratin 19. **(C)** Neoplastic nuclei positive for p63. **(D)** T-lymphocytes positive for CD5 labelling.

The biopsy specimen measured 1.7 × 1.2 cm. The hematoxylin and eosin (H&E) stained sections showed invasive, moderately differentiated squamous cell carcinoma involving skeletal muscle (Figure 2A). The neoplastic cells were disposed in sheets, nests and anastomosing cords. They were predominantly polygonal, but a minor spindle-shaped component was also present (Figure 2B). These cells had abundant eosinophilic cytoplasm with well-defined borders. Intercellular bridges were present but keratin production was not detected. The neoplastic nuclei were large, pleomorphic and contained vesicular chromatin and prominent nucleoli (Figure 2C). Scattered mitotic figures were present but necrosis was not evident.By immunohistochemistry, the tumor cells were positive for cytokeratins 5/6, 7, 19 and p63 (Figure 3A–C). Stains for cytokeratin 20, thyroglobulin, TTF1, synaptophysin, chromogranin, calcitonin, CD5 and p16 were negative (Figure 3D). Normal thyroid tissue or other histologic types of thyroid carcinoma in the biopsy specimen were lacking.

## Discussion

The diagnosis of thyroid PSCC is often achieved through a combination of clinical, radiologic, and ultimately histologic findings. Making the diagnosis can be challenging and some authors would believe that PSCC does not exist as an entity unto itself [2,4]. Clinically resembling anaplastic thyroid cancer, PSCC is extremely aggressive with median survival rates of less than six months often due to airway obstruction causing death [2,7,8]. With a one-month history of a neck mass coupled with a seventeen-day span between our patient’s presentation and death, we present the shortest median survival of all documented cases [4,9]. This abbreviated clinical course may be due to the absence of therapy, though PSCC cases lacking intervention possessed longer median survival periods [3,4,9]. While an immunocompromised state can accelerate neoplastic growth, this patient had a complete immune workup, and no deficiencies were detected. Presenting symptoms can include sudden onset of an enlarging mass, dysphagia, hoarseness, and pain, which were noted in the case presented [4,7]. Radiographically, PSCC often presents with advanced infiltration and effacement of neighbouring structures, which was noted in our case, given the invasion of the esophagus, displacement of the trachea and proximity to neck vasculature.

To further delineate thyroid PSCC from other differential diagnoses such as carcinoma showing thymus-like differentiation (CASTLE) and metastatic squamous cell, immunochemistry can be useful [2]. CASTLE tumors have been shown to exhibit CD5 expression, which our surgical specimen did not [2]. Moreover, the immunoprofiles of SCC often lack specificity, though CK7 and 19 have been shown to be positive in PSCC [2,6,9]. This was the case in our histological findings. Diagnosis of primary SCC was primarily supported by the histologic morphology of the biopsy. Given a negative calcitonin, medullary thyroid carcinoma diagnosis was also excluded [4,7]. As per Lam et al.’s findings, CK20 expression was negative, which refutes gastric, intestinal, or urothelial squamous cell carcinomas, which could potentially have metastasized to the thyroid [2,3,9]. Furthermore, in keeping with other thyroid PSCC cases in the literature, a negative TTF-1 and thyroglobulin helps to support the diagnosis of PSCC by excluding a cancer arising from follicular thyroid cells [2,4,10]. With the absence of radiographic evidence for a distant site of primary SCC, the supportive immunohistochemistry findings, and the unique morphology at the cellular level, we felt this presentation was most representative of a PSCC of the thyroid.

Interestingly, several weeks prior to presenting to our Emergency Department, a FNA biopsy performed in Ethiopia was suggestive of lymphocytic thyroiditis, which brings to light the debate of thyroid PSCC etiopathology. Two prominent concepts regarding the pathophysiology of PSCC exist including the “embryonic rest cell” and “metaplasia” theories [2,5,8,10,11]. Squamous cell derivation from remnants of the thyroglossal duct, the ultimobranchial body, and thymic epithelium is the basis of the “embryonic rest cell” theory [4,8,12]. The more accepted “metaplasia” theory, however, postulates the source of squamous cells to be from follicular, papillary or anaplastic cells [5,10,11]. The most common process of squamous metaplasia, Hashimoto’s Thyroiditis, has been associated with PSCC of the thyroid in four documented cases [1,10,11]. Given the patient’s previous FNA biopsy and the diagnosis of PSCC clinically, radiographically and histologically, this case likely aligns with previous cases of PSCC in the setting of lymphocytic thyroiditis, which makes this the fifth noted case in literature.

Unfortunately, there is no established treatment modality or combinations thereof to address PSCC, primarily due to the rarity of the presentation [2,4,12]. The tumor presented in this case was deemed unresectable due to invasion of the mass into neurovascular structures. Supportive measures were undertaken including a PEG tube, while chemoradiation was felt to not be feasible.

## Conclusions

We present the shortest documented median survival of primary squamous cell carcinoma of the thyroid gland in a thirty-six year-old male. The tumor’s morphology and immunoprofile were considered consistent with a primary neoplasm of the thyroid gland, arising in the background of lymphocytic thyroiditis. This case should build awareness of the aggressivity of the disease and the lack of established treatment options. Despite the rarity of this disease process, further discussion regarding diagnostic criteria discussion should be pursued.

## Consent

Written informed consent for publication could not be obtained from our deceased patient's next of kin despite all reasonable attempts. Every effort has been made to protect the identity of our patient and ensure anonymity.

## Competing interests

The authors declare that they have no competing interests.

## Authors’ contributions

JL drafted the manuscript. MK was involved in the the histopathologic analyses and also drafted the manuscript. SPC conceived of the study and also drafted the manuscript. All authors read and approved the final manuscript.
